# Longitudinal, Multi-Cycle Evaluation of Passive Function Improvement in People with Arm Spasticity Treated with Botulinum Toxin A

**DOI:** 10.3390/toxins18010051

**Published:** 2026-01-19

**Authors:** Stephen A. Ashford, Khan Buchwald, Klemens Fheodoroff, Jorge Jacinto, Ajit Narayanan, Richard J. Siegert, Christian Hannes, Lynne Turner-Stokes

**Affiliations:** 1 Department of Palliative Care, Policy and Rehabilitation, Cicely Saunders Institute, Florence Nightingale Faculty of Nursing, Midwifery and Palliative Care, King’s College London, London SE5 9RS, UK; lynne.turner-stokes@nhs.net; 2 Regional Hyper-Acute Rehabilitation Unit, Northwick Park Hospital, London North West University Healthcare, London HA1 3UJ, UK; 3 School of Clinical Sciences, Faculty of Health and Environmental Science, Auckland University of Technology, Auckland 0627, New Zealand; 4 Neurorehabilitation, Gailtal-Klinik, 9620 Hermagor, Austria; klemens.fheodoroff@me.com; 5 Centro de Medicina de Reabilitaçãode Alcoitão, Serviço de Reabilitação de adultos 3, 2649-506 Estoril, Portugal; 6 School of Engineering, Computer and Mathematical Sciences, Auckland University of Technology, Auckland 1010, New Zealand; 7 Department of Psychology and Neuroscience, Faculty of Health and Environmental Science, Auckland University of Technology, Auckland 0627, New Zealand; 8 Ipsen, 81677 Munich, Germany

**Keywords:** passive function, botulinum toxin, arm spasticity, goal attainment scaling, rehabilitation, upper limb spasticity, real-world evidence

## Abstract

Improvement in passive function (i.e., ease of caring for a limb) is a common goal for treatment of spasticity in the arm with botulinum toxin. A large international, observational, 2-year longitudinal study (ULIS-III, N = 953) was conducted in real-life practice. This original secondary analysis examines whether improvement in passive function goals were met over repeated injection cycles. We report changes by cycle measured by the Passive Function sub-scale of the Arm Activity measure (ArmA-PF) and examine predictors of improvement and injection occurrence. Inclusion in this analysis was based on passive function being selected as a primary or secondary goal for one or more cycle of treatment (*n* = 542/953). Goals were assessed at the start and end of each cycle using the Goal Attainment Test score and the ArmA-PF. Over all cycles of treatment, goals were set for 1641/2187 injections (75.0%) and achieved in 1250 (76.2%). Significant improvements in ArmA-PF score were identified for at least six cycles (*p* < 0.001) with evidence of cumulative benefit over successive cycles. This occurred regardless of patient-related baseline characteristics, with the possible exception of some relationship with injection localization techniques. In conclusion, repeated botulinum toxin injections provide significant improvement in passive function, which was sustained over repeated cycles of treatment.

## 1. Introduction

After a stroke or other acquired brain injury, many individuals experience impaired muscle control and difficulty with normal movement, and a substantial proportion develop disabling spasticity. Spasticity often develops following damage to the central nervous system and is marked by abnormal muscle overactivity [1]. This condition can cause stiffness and pain and may lead to contractures, joint deformities, and pressure ulcers [2]. These complications can significantly hinder functional activities and slow rehabilitation progress. Estimates of spasticity prevalence vary, but it has been reported in up to 42% of stroke survivors—equivalent to roughly 504,000 people in the UK [3,4,5].

Spasticity can interfere with the function of the body’s systems as well as activities that people want to perform. It can limit ‘active function’ (using the limb to perform functional tasks) or ‘passive function’ (the ability to care for the affected limb, such as maintaining hygiene or dressing) [6,7,8].

Injection with botulinum toxin type A (BoNT-A) is shown to be a safe and effective focal intervention for reducing spasticity [9,10,11,12], and is recommended for use in routine clinical practice by national and international guidelines [13,14,15].

BoNT-A therapy, when appropriately targeted and supported by complementary physical interventions, may enhance passive function by decreasing muscle spasticity, improving joint misalignment, and alleviating deformity-related strain [16]. When motor recovery is less likely, improving ease of care of the affected limb remains the predominant aim [17]. Previous studies have reported passive function as a major treatment goal for arm spasticity management [12,13,18,19,20,21,22,23,24,25].

Goal attainment scaling (GAS) is a structured approach for evaluating the degree to which individualized patient goals are achieved following treatment [26]. Detailed guidance on its application is covered extensively in the literature [27,28,29,30,31,32]. In complex interventions, patients often set multiple goals, with varying levels of achievement. The GAS T-score provides an aggregate measure of overall goal attainment; scores increasing from baseline indicate progress, and a score of 50 (SD ±10) reflects achievement as predicted. Values above 50 indicate outcomes exceeding expectations. GAS can assist, when categorized, in identifying patients whose treatment goals target specific functional domains. Although GAS is sensitive to changes during spasticity management [19,33,34], it measures goal achievement and has limitations regarding scaling and comparability [35]. Consequently, it should complement, not replace, standardized outcome measures [27].

In order to provide comprehensive assessment of both active and passive function in the context of interventions for arm spasticity, a standardized outcome measure needs to assess both of these constructs [36]. The Arm Activity measure (ArmA) was developed specifically for this purpose and includes two sub-scales addressing difficulty in active and passive function as separate entities. The ArmA [37] is a patient or carer-rated 20-item measure comprising a 7-item ‘passive function’ subscale (ArmA-PF, score range 0–28) and a 13-item ‘active function’ subscale (ArmA-AF, score range 0–52). Decreasing ArmA scores indicate a decrease in the difficulty of tasks and therefore an improvement in passive (or active) function. The ArmA has been systematically developed [38], supporting content validity. It has been psychometrically tested, providing evidence for construct validity, internal consistency, dimensionality, test–retest reliability, scaling, feasibility, and responsiveness [39].

The Upper Limb International Spasticity (ULIS) program consists of three international observational studies which uniquely describe and reevaluate real-life clinical practice in the use of BoNT-A to manage arm spasticity [40]. A primary element of the program is incorporation of GAS as the primary outcome measure, with validated measures including ArmA [41,42,43].

As a chronic condition, spasticity often requires ongoing intervention, and repeated BoNT-A injections have been shown to deliver long-term benefits across multiple symptoms [7,25,44,45,46,47,48]. ULIS-III [25,41], the third phase of the ULIS program, is among the largest multicenter studies assessing repeated BoNT-A treatment for upper-limb spasticity, with an effectiveness cohort of 953 patients. The study introduced the Upper Limb Spasticity Index (ULSI), integrating the Focal Spasticity Index (FSI) and recording GAS alongside selected standardized measures aligned with treatment priorities for each cycle. Results confirmed sustained treatment response over two years in goal attainment and functional outcomes—both passive and active [25]. Post hoc analyses suggested that injection guidance, female gender, and certain toxin types (favoring Abobotulinum over Onabotulinum) may predict better overall response [49].

This post hoc analysis aimed to examine outcomes from repeated BoNT-A injections in patients for whom passive function of the arm was explicitly identified as a treatment goal in routine clinical practice. The objectives were as follows:

(a) To assess whether goals for improving passive function were consistently achieved across multiple injection cycles over two years;
(b) To evaluate longitudinal changes (improvement) in passive function scores to determine any cumulative benefit on ease of limb care;
(c) To identify patient- and treatment-related factors associated with improvement following each injection.

Clinically, we hypothesized that patient-related predictors of improvement or increased injection frequency might include age, sex, time since injury, prior BoNT-A exposure, and spasticity severity or distribution. Intervention-related predictors were expected to include the number and location of muscles treated, total dose administered, and use of injection guidance techniques (e.g., electromyography, nerve stimulation, or ultrasound). We further hypothesized that increased number and location of muscles treated, greater total dose administered, and use of injection guidance may lead to a greater effect.

The clinical relevance is therefore in identifying ongoing responses for passive function treatment goals, with potential improvements for ease of care in people with arm spasticity. It was also important to establish other factors that contribute to a positive outcome in both practice and research.

## 2. Results

Within the ULIS-III effectiveness cohort (N = 953), 542 patients (56.9%) identified passive function as a treatment goal and were included in this analysis.

### 2.1. Demographics

As shown in Table 1, this subgroup did not differ significantly from others in terms of injury type or etiology but tended to be older (*p* = 0.002, adj *p* = 0.014) and have more chronic spasticity (*p* = 0.008, adj *p* = 0.058). They were more likely to exhibit regional spasticity (*p* = 0.026, adj *p* = 0.158), have prior BoNT-A exposure (*p* = 0.007, adj *p* = 0.058), and demonstrate higher baseline Disability Assessment Scale (DAS) scores (*p* < 0.001, adj *p* < 0.001) and greater distal Modified Ashworth Scale (MAS) scores (*p* < 0.001, adj *p* < 0.001), indicating more severe distal spasticity.

During the two-year period, patients in the passive function subgroup received 2187 injections, with 1641 (75%) associated with passive function goals. The proportion of such goals ranged from 64.8% to 82.6% in cycles 1–6, with achievement rates between 70.4% and 84.4%. For cycles 7–8, achievement ranged from 63.6% to 85.7%.

### 2.2. GAS-T- and ArmA-PF Score Analysis by Treatment Cycle

**Figure A1:**
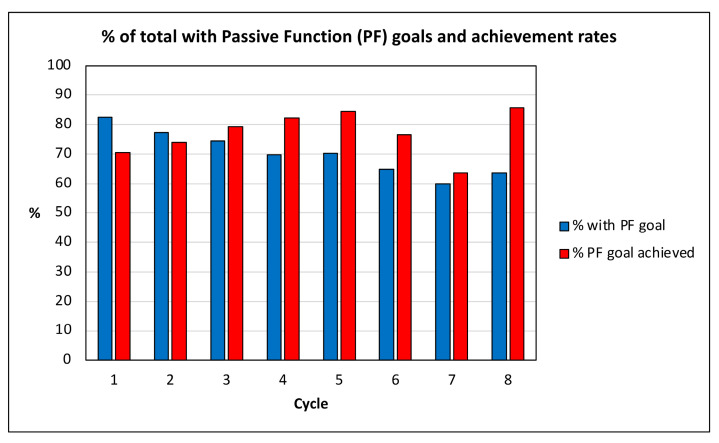
Analysis of passive function-related goals and achievement rates.

The first stage of analysis examined mean GAS T-scores and ArmA-PF scores across treatment cycles for patients with passive function goals and corresponding ArmA-PF data at the population level. In cycles 7 (*n* = 3) and 8 (*n* = 2), the samples became too small for statistical analysis, so our subsequent analyses focused on patients with up to 5–6 cycles of treatment with BoNT-A, depending on whether the numbers were large enough for analysis (see Appendix A Figure A1 and Table A1 for further information).

**Figure 1:**
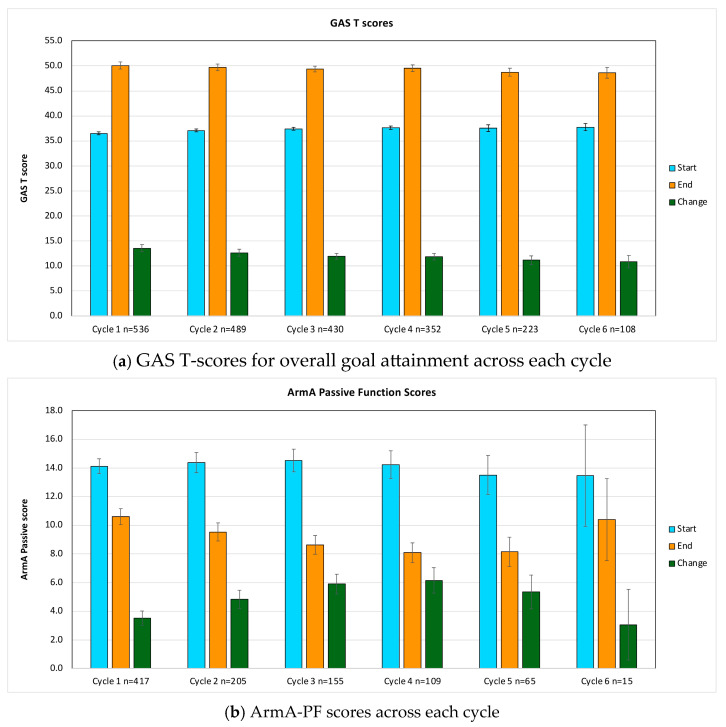
(**a**,**b**) present the mean GAS T-scores and ArmA-PF scores, including 95% bootstrapped confidence intervals, for all patients with passive function-related goals in any cycle (up to six cycles). Data are displayed for baseline, post-treatment, and the corresponding change per cycle. Note: *GAS* = goal attainment scaling.

Figure 1 illustrates mean pre- and post-treatment scores and change values (95% bootstrapped CI) for (a) GAS T-score in Table A1 and (b) ArmA-PF. Full numerical results and paired *t*-tests are shown in Table A2 (Appendix A), with *p*-values corrected for multiple testing via Holm–Bonferroni adjustment.

Across the first six cycles, GAS T-scores increased significantly (adjusted *p* < 0.001) and ArmA-PF scores decreased significantly (adjusted *p* = 0.034) (also see Appendix A Table A3), indicating sustained improvement in passive function and goal attainment. Mean GAS T-score changes remained >10 points, exceeding the Minimal Clinical Important Difference (MCID) threshold and confirming clinical significance.

ArmA-PF reductions increased from 3.5 points in cycle 1 to 6.1 in cycle 4, with smaller changes in later cycles, likely due to diminishing sample size. All cycles demonstrated clinically meaningful reductions (>3 points).

### 2.3. Serial Changes in Reported Difficulty with Passive Function by Number of Cycles

**Figure 2:**
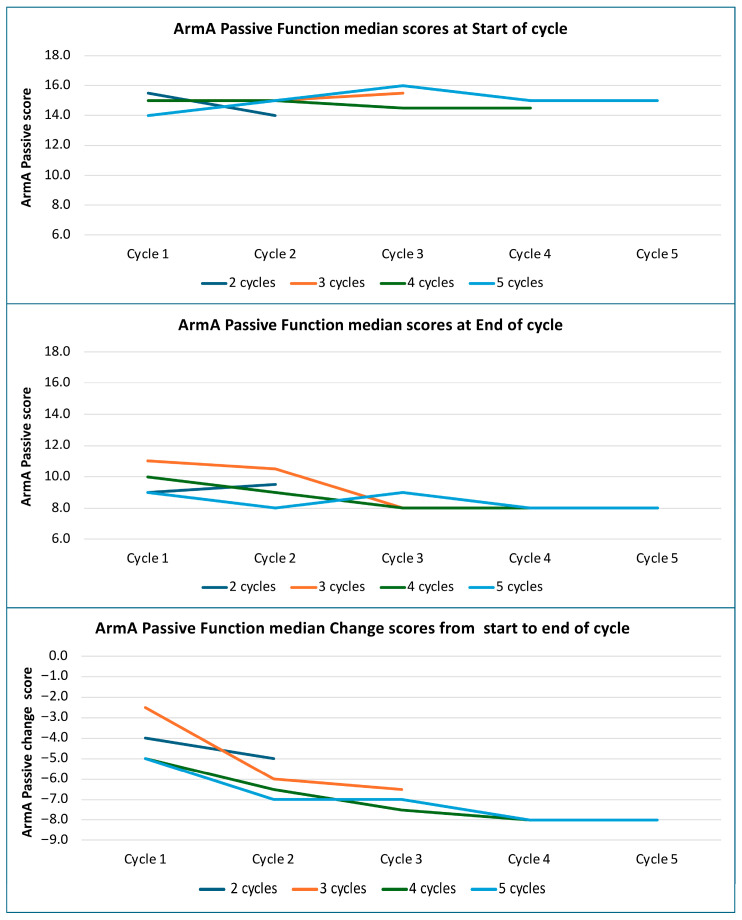
Median ArmA-PF scores by the number of cycles.

Figure 2 summarizes median ArmA-PF scores grouped by cycle count for patients completing up to five cycles, since no patients with six cycles had an ArmA-PF score in every cycle. The initial step in this exploratory analysis involved visual inspection of the graphical data to identify trends and validate subsequent statistical testing. Baseline scores remained stable, while end-of-cycle scores declined progressively, with greater reductions observed in cycles 1–4 and sustained thereafter. For patients completing four or five cycles, median change improved from −5 points in cycle 1 to −8 points in cycles 4 and 5.

Mixed effect models demonstrated a small but significant trend towards improvement over successive cycles observed at both the start and end of each cycle for ArmA-PF scores (*p* < 0.001, adj *p* < 0.001) (see Table 2), indicating some cumulative effect from repeated injections.

While this initial analysis excludes consideration of other covariates (see Table 2), we aimed to explore potential patient- and treatment-related characteristics associated with greater improvement in passive function.

### 2.4. Predictors of Improvement in Passive Function Scores

To explore predictors of passive function improvement, a mixed-effects model examined baseline patient-related factors, defined by reductions in ArmA-PF scores from cycle start to end (see Table 3). The considered variables included age, sex, neurological injury type and etiology, time since onset, previous BoNT-A treatment, and spasticity severity/distribution. None of these appeared to have a significant effect on improvement in passive function in this cohort, with the possible exception of more severe spasticity in the hand and wrist (as demonstrated by higher distal MAS scores) (*p* = 0.001, adj *p* = 0.008). In this instance, analysis of ArmA-PF outcomes showed that patients with more severe spasticity at baseline were able to achieve better passive function results at the end of each cycle.

A further mixed effects model was used to examine treatment-related factors associated with improvement in passive function including the frequency of injection across the study (cycle number), the number of muscles injected, the location of injections (shoulder, arm, forearm, and hand muscles), whether injection guidance was used, and the total dose of BoNT-A (as indicated by the proportion of maximum recommended total dose used in each cycle).

Factors that were significantly associated with greater improvement in ArmA-PF scores were higher doses of BoNT-A (B = −0.02; *p* = 0.025) and the use of injection guidance (B −1.16; *p* = 0.011), indicating a significant decrease in ArmA-PF scores in end-of-cycle compared to start-of-cycle scores when using injection guidance or increasing the dose administered, although these were non-significant when using adjusted *p*-values, shown in Table 4. There was no significant relationship between the magnitude of change in the ArmA-PF score and the number of injection cycles when other treatment-related factors were included in the model.

### 2.5. Impact of Cycle Number and Treatment Variables on Start- and End-of-Cycle Scores

Mixed effects analyses, summarized in Table 5 and Table 6, examined the influence of cycle number and treatment variables on ArmA-PF scores at both the beginning and end of each cycle. Results demonstrated a modest yet statistically significant decline in ArmA-PF scores with each additional cycle, amounting to −0.29 (adj *p* < 0.001) at cycle start and −0.32 (adj *p* < 0.001) at cycle end, suggesting a cumulative therapeutic effect. Higher ArmA-PF scores before injection were also statistically associated with higher doses of BoNT-A (Adj *p* < 0.001), amounting to 0.04 for every 1 percent of the maximum dose of BoNT-A given.

## 3. Discussion

This post hoc evaluation of the outcomes gathered in the ULIS-III study assessed the sustained impact of repeated BoNT-A injections on passive function—defined as ease of caring for the affected arm—within routine clinical practice. The findings confirm that improving passive function was a key treatment objective for over half of the patients and that repeated administrations provided ongoing benefit throughout a two-year observation period. From a clinical perspective it is important to re-emphasize the importance of passive function gain as an aim for treatment so that clinicians continue to prioritize this. It is also important to understand that increasing gain in passive function/ease of care is clinically possible and likely to happen with repeated cycles of treatment.

### 3.1. Response to Repeated Injections

Over at least six treatment cycles, passive function improved significantly between the start and end of each treatment cycle, as measured by a reduction in ArmA-PF scores and shown by goal attainment. This reduction occurred regardless of baseline characteristics such as age, type of neurological damage, or chronicity of spasticity, other than a trend towards less improvement with more severe spasticity in the hand and wrist. In other words, it did not matter how old the patient was, the nature of their injury, or how long they had had spasticity—BoNT-A injection still made it significantly easier to care for the affected arm after each injection and improved passive function.

More severe spasticity in the hand and wrist (as demonstrated by higher distal MAS scores at baseline) (*p* = 0.001) seemed to be associated with greater improvements in passive function as measured by ArmA-PF at the end of the cycle. This makes clinical sense in that, when spasticity is more severe, there is more potential for change with BoNT-A administration, and thus greater improvement in passive function than in milder spasticity. Although no longer significant after adjustment of *p*-values, there was also a statistical trend towards greater improvement with higher doses of BoNT-A and use of injection guidance, which is in line with the direction that we hypothesized and resonates both with clinical experience and other published findings (see below).

### 3.2. Cumulative Benefit from Repeated Injections

Amongst the patients who had between two and six injections during the study period, there was evidence indicative of a small cumulative effect on improvement in passive function, which carried on over successive cycles for ArmA-PF scores at the start and end of each cycle (*p* < 0.001). This is a unique finding which has not, to our knowledge, been previously shown. It appeared to be unrelated to other treatment factors, suggesting that this may be a real effect of the repeated cycles of treatment rather than a sampling error, and therefore warrants further investigation to determine if this is indeed a true effect.

From a clinical perspective, this possible cumulative effect on ease of caring for the limb would not be surprising, as it resonates with clinical experience. There are a number of possible explanations for it, which include (a) the potential for spasticity to stabilize and its expression to reduce over time [50], (b) the effect of ongoing concomitant physical interventions (such as splinting and positioning), which provide an ongoing stretch to muscle and soft tissues resulting in decreased contracture and limb deformity (i.e., increased muscle length due to stretch to connective tissue and increase in sarcomeres) [50,51,52,53,54,55], (c) a learning effect on the part of the injector and treating team, enabling them to target the most affected/responsive muscles, localize them with injection guidance techniques, and using higher doses of BoNT-A where appropriate, resulting in the impact of better spasticity reduction.

Higher ArmA-PF scores before injection were also statistically associated with higher doses of BoNT-A (*p* < 0.001), which again resonates with clinical experience. Patients presenting with more severe difficulties with passive function are likely to require higher doses of BoNT-A to manage the problem.

### 3.3. Comparison of Findings with Other Studies

Our findings align with previous research, which consistently identifies passive function—defined as ease of caring for the upper limb—as a primary therapeutic goal, particularly when the hand or arm is non-functional [12,48,56,57]. While fewer studies have examined the longitudinal impact of repeated BoNT-A injections on arm spasticity, those that have have also demonstrated a continuing benefit from repeated injections [58] and the importance of injection guidance in the response to treatment.

We are not aware of any previous study reporting a cumulative effect of repeated injection of BoNT-A on ease of caring for a limb, but a previous secondary analysis of the primary ULIS-III longitudinal study revealed evidence of cumulative benefit from repeated injections in the management of spasticity-related pain [58]. Therapy inputs were also found to be important [49], and this has been further supported in a worldwide survey of the perception of clinicians engaged in the management of spasticity [59].

Provision of physical therapy inputs and incorporation of spasticity management into a holistic program of rehabilitation or management has been emphasized in a number of national and international guidelines for focal spasticity intervention [15,49,59,60]. Spasticity management using botulinum toxin is therefore not an isolated intervention and should be viewed in the context of the overall rehabilitation program [61,62]. In planning treatment, the wider rehabilitation or care context is particularly important to consider when the goal of treatment is passive function- or care-related in nature.

### 3.4. Important Messages for Clinicians Managing Arm Spasticity

With a specific clinical focus, it is important to re-emphasize the importance of passive function gain as an aim for treatment so that clinicians continue to prioritize this in treatment planning and delivery. It is also important to understand that increasing gains in passive function/ease of care is clinically possible and likely to happen with repeated cycles of treatment, with maintenance of some benefit over those cycles.

The findings provide important justification to support repeated BoNT-A injection as part of ongoing management to maintain ease of caring for arm spasticity and passive function gain [18,20].

For a proportion of people, ongoing administration (three or more cycles per year) of BoNT-A will be required to sustain the benefits over a longer time period, often critically preventing deterioration in their condition and possibly also supporting longer-term improvements. However, concomitant physical management is also likely to be necessary in order to maintain long-term benefits.

These benefits are likely to be achieved irrespective of patient-related characteristics, including age, gender, time since injury, previous injection with BoNT-A, and severity/distribution of spasticity, but they may be enhanced through optimal injection techniques such as guidance and total dose of BoNT-A used.

There are challenges with long-term adherence to physical management and, indeed, repeated cycles of BoNT-A administration. This paper does not aim to address issues of self-efficacy and treatment engagement specifically. However, the goal-setting process used (incorporating goal attainment scaling), by ensuring patient/participant engagement, partially aids in achieving ‘buy-in’ for associated rehabilitation and management.

### 3.5. Strengths and Limitations

Several strengths and limitations warrant consideration.

 **1.**  **Strengths** include its large sample size and the fact that it was conducted within real-world clinical practice, encompassing broad international representation, diverse etiologies, and all BoNT-A formulations, thereby enhancing the generalizability of findings.
 **2.**  **Limitations** relate primarily to the absence of a control group—an inherent feature of the longitudinal design—and variability in site distribution across countries, which may limit representativeness in certain settings. Additional sources of bias may include clinician expertise, injector skill, and external prescribing constraints that restrict injection frequency.

The ULIS-III dataset provides a robust foundation for post hoc analyses; however, such analyses inherently increase the risk of type I error due to multiple testing. In this study, we specifically examined the influence of patient characteristics (e.g., age, gender, severity, and chronicity) on passive function outcomes following repeated injections, applying statistical methods to minimize this risk. However, in so doing, there is some risk of obscuring important findings or increasing type II error, though this is addressed already in the paper related to overall treatment dose and injection guidance. This sub-analysis focuses only on patients with goals for passive function and needs to be read in conjunction with other publications from the ULIS III study, including the main results paper (25). Other complementary sub-analyses for different key goal areas, such as pain, which is published in *Toxins* (58), and further analyses on goal attainment and active function, which are in progress, should also be referred to.

## 4. Conclusions

In spite of this study’s limitations, the results indicate that improving ease of care for the spastic arm is a prevalent treatment goal and that repeated injections result in sustained benefits.

## 5. Materials and Methods

### 5.1. Study Design and Participants

This retrospective analysis utilized data from ULIS-III (NCT02454803), a large-scale, international, prospective observational study, previously described in detail [25,41].

In summary, ULIS-III followed adults living with arm spasticity over a two-year period in real-world clinical settings, assessing integrated care programs incorporating both BoNT-A administration and physical intervention. The study was conducted across 58 sites in 14 countries, spanning 4 continents.

Up to 30 eligible adult patients (≥18 years old) for whom BoNT-A injections had been planned to treat arm spasticity were consecutively recruited per center. Treatment complied with local regulatory requirements and standard practice, using any approved BoNT-A product.

Patients were monitored for two years, during which multiple treatment cycles occurred. Treatment was goal-directed, with flexibility to adjust primary and/or secondary goals at the start of each cycle. Ethical approval and written informed consent for anonymized data collection were obtained as required.

### 5.2. Outcome Assessment and Measures

Baseline and follow-up assessments were conducted throughout the two-year study using the Upper Limb Spasticity Index (ULSI), which combines GAS with selected standardized measures aligned to the priority treatment goal areas for each cycle.

Goal setting and attainment were evaluated using the Goal Attainment Scaling Evaluation of Outcome for Upper Limb Spasticity Tool [41], employing the GAS light method [27] to calculate GAS T-scores. Patients could define up to three goals per cycle (one primary, two secondary). A mean GAS T-score of 50 (SD +/− 10) indicates expected goal achievement [26], and a 10-point increase from baseline represents the minimal clinically important difference [30].

The Arm Activity measure (ArmA) is a validated patient-reported outcome measure of perceived difficulty in both passive and active arm function in individuals with hemiparesis [38,39]. It comprises 20 items, each scored on a 5-point Likert scale ranging from 0 (no difficulty) and 4 (unable to do task). It has two subscales, a 7-item scale of passive function (ease of caring for the spastic arm) score with a range of 0–28, and a 13-item scale of active function (using the limb for functional tasks) score with a range of 0–52. Higher scores on the ArmA measures equal greater impairment; therefore, a negative change score indicates improvement over time.

This secondary analysis focused on patients within the effectiveness population (*n* = 953) who identified ease of care for the affected limb (passive function) as a treatment goal during the study period.

### 5.3. Statistical Analysis

Patients in the ‘passive function subgroup’ set at least one goal—primary or secondary—related to improving passive function during any treatment cycle. Each cycle had two assessments, pre-injection, and post-injection, change in passive function was quantified by the difference in ArmA-PF scores between both assessment timepoints.

Given the observational nature of ULIS-III and delivery in routine clinical practice, patients with passive function goals did not necessarily maintain such goals across all cycles, which complicated the longitudinal evaluation of data. Accordingly, initial evaluations involved descriptive and visual exploration of trends in the data, followed by more advanced statistical modeling.

Although up to eight cycles were recorded, very few patients with relevant data (*n* = 2 and *n* = 3, respectively), such as goals related to passive function and before and after ArmA-PF scores, completed seven or eight cycles, so for the primary analysis, we included the results for up to a maximum of six cycles.

Variables in this dataset were analyzed without any transformation, and no outliers were excluded. Missing values were accounted for in the mixed effects models using the random forests algorithm in the R package missForest (version 1.5), using all variables included in the mixed effect models only. There were three imputations for cycle in this study, one for re-injection scores, one for post-injection scores, and one for change scores. Because the ArmA-PF score was the response variable, we removed missing values in the ArmA-PF score before each imputation. This maximized the valid sample size without needing to impute the response variable. No other imputation was implemented in this study. Holm’s method was applied to adjust *p*-values for multiple comparisons [63]. This method is a standard, less conservative, uniformly more powerful method to adjust *p*-values compared to the Bonferroni correction [64]. Nevertheless, it is recognized that adjustment of *p*-values can potentially obscure more subtle trends arising from the analysis, so where relevant, we have included both the adjusted and non-adjusted *p*-values.

Analyses were conducted in R (version 4.3.2). The primary objective of this study included evaluating changes in GAS T-scores and ArmA-PF scores across repeated cycles for patients with passive function goals. We first ran statistical tests to identify if the demographics in the effectiveness population were significantly different to those with at least one ArmA-PF score. Population-level analyses involved calculating mean pre, post, and change scores per cycle with 95% confidence intervals (CIs) and comparing change scores using paired *t*-tests stratified by cycle number (see Table A2 in Appendix A). To capture change over repeated cycles at the individual level, we present descriptive charts of the median ArmA-PF score grouped by the maximum number of cycles each individual had, up to five cycles (as none of the patients with a maximum of six cycles had complete ArmA-PF scores in every cycle).

Mixed effects models assessed predictors of start-of-cycle scores, end-of-cycle scores, and change-in-ArmA-PF scores, while controlling for the confounding effect of repeated measures. We conducted two analyses for change in cycle scores, one for the effect of baseline characteristics on change and one for the effect of treatment characteristics on change. We also conducted two analyses each for both the start-of-cycle scores and end-of-cycle scores: one using cycle number only and one using cycle number and other treatment characteristics. Observations were at the level of the patients and cycle number, rather than the patients alone. Because of the AB design and the maximum of five cycles, as well as small sample sizes in later cycles, observations were not independent. To account for this dependency, a random effect term for each patient was incorporated. This modeling strategy improved sensitivity for detecting significant differences between the predictors and outcome variables.

Based on prior analyses [34,49], we proposed that reductions in passive function might be influenced by patient or intervention-related factors such as age, gender, chronicity of spasticity (years since onset), previous treatment with BoNT-A, baseline severity, and distribution of spasticity (as measured by the composite MAS for the proximal (elbow and shoulder) [25] and distal (hand, wrist, and finger) muscles, and the number of cycles (or visits) required during the 2-year period of the study. We also hypothesized that intervention-related factors may include the number of muscles injected, the part of the arm injected, the total dose injected, and the use of techniques to guide injection (e.g., using electromyography, muscle stimulation, or ultrasound). As the study included any of the three licensed preparations of BoNT-A (Abobotulinum Toxin, Incobotulinum Toxin, and Onabotulinum Toxin), which have different units, the total dose used was expressed as a percentage of the maximum recommended dose for each product, referencing the UK Summary of Product Characteristics.

## Figures and Tables

**Table 1:** Demographics of the overall effectiveness population and the passive function subgroup.

	Effectiveness Population (N = 953)		Passive Functionsubgroup (N = 542)		Patients Without Passive Function Goals (N = 411)		*p* Value	Adjusted *p* Value
Demographic	N	%	N	%	N	%	*p*	Adj *p*
Sex–*n*, %							0.362	1
Male	537	56.3	298	55.0	239	58.2		
Female	416	43.7	244	45.0	172	41.8		
Age–*n*, %							0.002	0.014
<51 years	364	38.2	181	33.4	183	44.5		
51–60 years	254	26.7	151	27.9	103	25.1		
>60 years	335	35.2	210	38.7	125	30.4		
Type of injury–*n*, %							0.069	0.347
Acquired brain injury	870	91.3	504	93	366	89.1		
Spinal cord injury	15	1.6	7	1.3	8	1.9		
Congenital	44	4.6	17	3.1	27	6.6		
Progressive neurological	20	2.1	12	2.2	8	1.9		
Other	4	0.4	2	0.4	2	0.5		
Etiology of Acquired brain injury *n*, %							0.585	1
Vascular (stroke)	786	90.3	455	83.9	331	80.5		
Trauma	71	8.2	40	7.4	31	7.5		
Hypoxic	25	2.9	12	2.2	13	3.2		
Tumor	19	2.2	9	1.7	10	2.4		
Inflammatory/infective/other	52	6.0	26	4.8	26	6.3		
Time since onset of injury							0.008	0.058
<1 year–*n*, %	145	15.2	68	12.5	77	18.7		
>1 year–*n*, %	769	80.7	455	83.9	314	76.4		
Years–Mean (95% CI)	7.6		7.5	(6.7, 8.3)	7.7	(6.7, 8.7)	0.790	1
Missing–*n*, %	39	4.1	19	3.5	20	4.9		
Previous injections with BoNT-A for arm spasticity–*n*, %							0.007	0.058
No (Naïve)	318	33.4	161	29.7%	157	38.2%		
Yes (Non-Naïve)	635	66.6	381	70.3%	254	61.8%		
Distribution of spasticity–*n*, %							0.026	0.158
Focal	190	19.9	94	17.3	96	23.4		
Regional	763	80.1	448	82.7	315	76.6		
Total	953	100	542	100	411	100		
Baseline function/spasticity	Mean	95% CI	Mean	95% CI	Mean	95% CI	*t*-test	
Baseline DAS total score	6.1	(5.9, 6.3)	6.5	(6.3, 6.7)	5.6	(5.3, 5.8)	<0.001	<0.001
Proximal composite MAS score	3.7	(3.6, 3.8)	3.7	(3.6, 3.8)	3.7	(3.5, 3.8)	0.578	1
Distal composite MAS score	6.1	(5.9, 6.2)	6.4	(6.2, 6.5)	5.7	(5.4, 5.9)	<0.001	<0.001

DAS = Disability Assessment Scale, MAS = Modified Ashworth Scale; CI = bootstrapped confidence interval. χ^2^ = Chi square.

**Table 2:** Mixed effects model of cycle number predicting start and end of ARMA-PF scores.

Variable	B	SE	df	T	*p*	Adjusted *p*
Start of Cycle						
Intercept	13.90	0.24	588.18	57.22	<0.001	<0.001
Cycle number	−0.28	0.08	532.59	−3.63	<0.001	<0.001
End of cycle						
Intercept	10.60	0.24	659.59	43.72	<.0001	<0.001
Cycle number	−0.32	0.06	1059.03	−5.06	<0.001	<0.001

**Table 3:** Mixed effects model of baseline characteristics and change in ArmA-PF score.

Variable	B	SE	df	T	*p*	Adjusted *p*
Intercept	−0.81	1.03	694.62	−0.79	0.429	1
Sex						
Female	−0.57	0.43	504.20	−1.33	0.184	1
Age						
51–60	−0.02	0.56	514.16	−0.04	0.968	1
>60	−0.59	0.52	503.30	−1.14	0.253	1
Type of injury						
Not acquired brain injury	−1.99	1.03	503.37	−1.93	0.054	0.538
Etiology						
Not vascular	0.71	0.72	510.59	0.98	0.329	1
Years since injury	0.03	0.03	522.42	1.26	0.208	1
Previous treatment with botulinum toxin						
Yes	0.44	0.48	506.63	0.92	0.357	1
Distribution of spasticity						
Regional	−0.04	0.59	553.63	−0.07	0.947	1
MAS Proximal	−0.07	0.17	913.38	−0.42	0.674	1
MAS Distal	−0.39	0.11	900.55	−3.39	0.001	0.008

Note. Up to cycle 5 only.

**Table 4:** Mixed effects model of change for ArmA-PF scores, with individual random effects. A. mixed effects model of treatment characteristics and change in ArmA-PF score.

Variable	B	SE	df	T	*p*	Adjusted *p*
Intercept	0.95	0.89	922.04	1.07	0.284	1
Cycle number	−0.15	0.09	622.24	−1.69	0.092	0.459
Number of muscles injected	−0.21	0.11	863.15	−1.95	0.051	0.357
Injection guidance used						
Yes	−1.16	0.45	749.03	−2.56	0.011	0.095
Shoulder injected						
Yes	0.32	0.38	878.21	0.83	0.407	1
Arm injected						
Yes	−0.81	0.43	932.95	−1.87	0.062	0.370
Forearm injected						
Yes	−0.68	0.76	914.75	−0.90	0.369	1
Hand injected						
Yes	0.31	0.37	933.35	0.84	0.401	1
Proportion of max dose	−0.02	0.01	784.31	−2.25	0.025	0.200

Note. Up to cycle 5 only.

**Table 5:** Mixed effects model of cycle number and treatment variables, associated with ArmA-PF scores at the start of each cycle.

Variable	B	SE	df	T	*p*	Adjusted *p*
Intercept	10.90	0.65	924.66	16.74	<0.001	<0.001
Cycle number	−0.29	0.07	529.17	−3.94	<0.001	<0.001
Number of muscles injected	0.11	0.09	968.93	1.23	0.220	0.440
Injection guidance used						
Yes	0.01	0.46	917.22	0.02	0.988	0.988
Proportion of max dose	0.04	0.01	959.25	4.12	<0.001	<0.001

**Table 6:** Mixed effects model of cycle number and treatment variables, associated with ArmA-PF scores at the end of each cycle.

Variable	B	SE	df	T	*p*	Adjusted *p*
Intercept	11.56	0.61	1195.43	18.83	<0.001	<0.001
Cycle number	−0.32	0.06	1055.18	−4.93	<0.001	<0.001
Number of muscles injected	−0.09	0.09	1381.98	−1.07	0.285	0.569
Injection guidance used	−0.89	0.46	1072.57	−1.94	0.052	0.157
Yes						
Proportion of max dose	<0.01	0.01	1348.30	0.55	0.582	0.582

## Data Availability

Data sharing is restricted due to the limits of patient confidentiality and consent. Further details on Ipsen’s sharing criteria, eligible studies, and process for sharing are available here: https://vivli.org/members/ourmembers/, accessed on 10 January 2026. Any requests should be submitted to www.vivli.org (accessed on 10 January 2026) for assessment by an independent scientific review board.

## Appendix A. List of Tables and Figures

**Table A1:** Analysis of passive function-related goals and achievement rates.

Cycle	Total N in Sample	N (%) with a PF Goal	N (%) with PF Goal Achieved
1	536	443 (82.6%)	355 (80.1%)
2	489	378 (77.3%)	319 (84.4%)
3	424	315 (74.3%)	269 (85.4%)
4	348	243 (69.8%)	215 (88.5%)
5	219	154 (70.3%)	133 (86.4%)
6	105	68 (64.8%)	58 (85.3%)
7	55	33 (60.0%)	23 (69.7%)
8	11	7 (63.6%)	6 (85.7%)
Total	2187	1641 (75.0%)	1250 (76.2%)

Footnote: Up to 8 cycles are included in this figure but not in the analysis in the paper due to the small participant numbers in cycles 7 and 8.

**Table A2:** Paired GAS T-scores and ArmA-PF scores across the 6 cycles with 95% CIs.

	Start of Cycle			End of Cycle			Change			Paired *t* Tests			
Cycle	N	Mean	95% CI	N	Mean	95% CI	N	Mean	95% CI	t	df	*p*	Adjusted *p*
GAS T-score													
1	414	36.4	36.1, 36.7	414	50.0	49.2, 50.8	414	13.6	12.8, 14.4	33.9	413	<0.001	<0.001
2	204	36.6	36.1, 37.0	204	50.6	49.6, 51.5	204	14.0	12.9, 15.0	26.8	203	<0.001	<0.001
3	155	37.1	36.7, 37.5	155	50.8	49.9, 51.8	155	13.8	12.7, 14.8	26.5	154	<0.001	<0.001
4	109	37.7	37.2, 38.1	109	52.0	50.9, 53.0	109	14.3	13.1, 15.4	24.9	108	<0.001	<0.001
5	65	37.7	37.0, 38.4	65	49.8	48.3, 51.2	65	12.0	10.6, 13.5	16.2	64	<0.001	<0.001
6	15	37.9	36.1, 39.6	15	48.8	46.3, 51.2	15	10.9	8.1, 13.7	8.2	14	<0.001	<0.001
ArmA-PF scores													
1	414	14.1	13.6, 14.6	414	10.6	10.0, 11.1	414	−3.5	−4.0, −3.0	−13.5	413	<0.001	<0.001
2	204	14.4	13.6, 15.1	204	9.5	8.8, 10.1	204	−4.9	−5.6, −4.3	−14.9	203	<0.001	<0.001
3	155	14.5	13.7, 15.4	155	8.6	7.9, 9.3	155	−5.9	−6.6, −5.2	−16.8	154	<0.001	<0.001
4	109	14.2	13.2, 15.2	109	8.1	7.4, 8.8	109	−6.1	−7.0, −5.3	−13.9	108	<0.001	<0.001
5	65	13.5	12.2, 14.9	65	8.2	7.1, 9.2	65	−5.4	−6.6, −4.1	−8.8	64	<0.001	<0.001
6	15	13.5	9.3, 17.6	15	10.4	7.2, 13.6	15	−3.1	−5.9, −0.3	−2.3	14	0.034	0.034

t = *t* test statistic; df = degrees of freedom; *p* = *p* value; GAS = goal attainment scaling.

**Table A3:** Median ArmA passive scores at the start of each cycle, grouped by the number of repeated cycles each patient.

	Median ArmA Passive Score by Number of Cycles			
Start-of-cycle scores	2 cycles *n* = 32	3 cycles *n* = 38	4 cycles *n* = 59	5 cycles *n* = 57
Cycle 1	15.5	15.0	15.0	14.0
Cycle 2	14.0	15.0	15.0	15.0
Cycle 3		15.5	14.5	16.0
Cycle 4			14.5	15.0
Cycle 5				15.0
End-of-cycle scores	2 cycles *n* = 32	3 cycles *n* = 38	4 cycles *n* = 59	5 cycles *n* = 57
Cycle 1	9.0	11.0	10.0	9.0
Cycle 2	9.5	10.5	9.0	8.0
Cycle 3		8.0	8.0	9.0
Cycle 4			8.0	8.0
Cycle 5				8.0
Change scores	2 cycles *n* = 19	3 cycles *n* = 17	4 cycles *n* = 40	5 cycles *n* = 32
Cycle 1	−4.0	−2.5	−5.0	−5.0
Cycle 2	−5.0	−6.0	−6.5	−7.0
Cycle 3		−6.5	−7.5	−7.0
Cycle 4			−8.0	−8.0
Cycle 5				−8.0
